# Problem gambling and psychological distress: a cross-national perspective on the mediating effect of consumer debt and debt problems among emerging adults

**DOI:** 10.1186/s12954-018-0251-9

**Published:** 2018-09-03

**Authors:** Atte Oksanen, Iina Savolainen, Anu Sirola, Markus Kaakinen

**Affiliations:** Faculty of Social Sciences, University of Tampere, 33014 Tampere, Finland

**Keywords:** Gambling, Young adults, Psychological distress, Debt, Credit, Default

## Abstract

**Background:**

Severe economic difficulties are common among younger generations who currently have an easy access to consumer credit and payday loans in many Western countries. These accessible yet expensive short-term loans may lead to more severe financial difficulties, including default and debt enforcement, both which are defined as debt problems within this study. This study hypothesized that consumer debt and debt problems mediate the relationship between problematic gambling and psychological distress. Excessive gambling can be funded with consumer debt, which in turn leads to the accumulation of financial stressors and, eventually, psychological distress.

**Methods:**

Three studies were conducted to examine the hypotheses. Study 1 used a demographically balanced sample of Finnish participants aged 18 to 25 years (*n* = 985, 50.76% female). Study 2 used a sample collected from Finnish discussion forums and social networking sites, with participants ranging from 18 to 29 years of age (*n* = 205, 54.63% female). Study 3 used a demographically balanced sample of American youths aged 18 to 25 years (*n* = 883, 50.17% female). Analyses were based on generalized structural equation models examining the role of problem gambling, consumer debt, and debt problems (i.e., default and debt enforcement) on psychological distress. Additional mediation analysis was run with treating both instant loans and debt problems as mediators.

**Results:**

All three studies showed that problem gambling was associated with consumer debt, which was further associated with debt problems. Both consumer debt (studies 1 and 2) and debt problems (study 3) were associated with psychological distress. Problem gambling was also directly associated with psychological distress in studies 1 and 3, but not in study 2. In Finland, consumer debt mediated the relationship between problem gambling and psychological distress (studies 1 and 2), while study 3 underlined the mediating role of debt problems in the USA, where consumer debt itself was not positively associated with psychological distress.

**Conclusions:**

The results of the three studies indicate that problem gambling-related psychological distress is partly explained by consumer debt. Consumer credit and payday loans may provide resources for gamblers that enable them to keep up with the habit. This may eventually lead to debt problems and psychological distress. Cross-national differences exist, but in both Nordic and American models, similar mechanisms prevail. The results imply that limiting consumer debt among emerging adults could cushion the financial and psychological costs of problem gambling.

## Background

Young people of today are taking debt at unprecedented levels [1–3]. General deregulation has made consumer lending attractive for various financial institutions throughout Western countries, including predatory and fringe banking agencies [4, 5]. To this effect, the current young generation has been even labeled as a “generation indebted” in the USA [2]. In many OECD countries, young people are provided with easy access to consumer credit and payday loans once they turn 18 years of age. These loans typically have high interest rates and they must be paid back in a relatively short period of time, when compared to long-term loans, such as student loans or mortgages [1, 6]. Payday loans are also accessible for those population groups who do not have stable income or assets to obtain loans with lower interest rates [1, 7].

New types of opportunities for money lending and consumer credit have been considered as a major cause of financial difficulties among younger generations, leading to debt default and debt enforcement [1, 8, 9]. The current generation of American young people is relying on unsecured debt, unlike their predecessors in earlier generations [2]. They also use more credit and repay loans that come with lower rates, compared to older generations [10]. Default, debt problems, and insolvency are surprisingly common phenomena, especially among young people between the ages 18 and 25 who are still learning financial independence and management [11]. This period of early or “emerging” adulthood is characterized by instability, rapid life changes, and explorations [12]. Financial risk taking is also manifested at this age [9, 13, 14], but due to young age and inexperience, emerging adults do not yet have substantial assets to cushion the adverse effects [15, 16], despite the potential financial support received from the parents [17, 18].

The current rise of consumer debt coincides with the global rise of gambling opportunities. Gambling is a form of potential risk behavior which can manifest even at a relatively young age. New gambling technologies, such as various online gambling platforms, have provided extended and convenient opportunities for young people to gamble practically without any age restrictions [19–23]. Gambling has also increased its popularity as a recreational activity in many countries [24, 25], particularly among youth [22]. Moreover, young people of the present day are living in a time where gambling is widely promoted and advertised both offline and online. These types of encouraging affirmations provided by the society may foster positive attitudes towards gambling among young individuals [26].

Gambling has consistently been shown to co-occur with, and may even lead to, other behavioral problems among some young people. Prevalence of problem gambling reported in studies ranges from 0.2 to 12.3% among adolescents and young people aged 10 to 24 [22]. Potentially harmful effects of gambling on young people specifically include social problems, poor school performance [27, 28], and psychological distress [29, 30]. Problem gambling also has a high comorbidity with other psychological disorders, such as alcohol and substance abuse, as well as mood and anxiety disorders, and depression and internalizing disorders in general [31]. Longitudinal studies have shown that problem gambling increased the odds of mood disorders, generalized anxiety disorder, posttraumatic disorder, alcohol use disorders, and alcohol dependence 3 years later, even after adjustment for a number of sociodemographic and medical factors and life events [32].

Consumer credit and different types of loans can be highly attractive for those who gamble as they provide access to additional financial resources 24 h/7. Serious financial hardship is one of the negative long-lasting consequences of gambling [24, 33]. Excessive gamblers in particular begin to chase the win and may then end up losing even more [34, 35]. Due to these reasons, poor financial judgments, such as taking a loan or borrowing money with high interest rates, are part of the gambling pathology. The DSM-5, for example, refers to ways in which gamblers search for opportunities to gain money in order to gamble [36]. Studies have shown that access to money is central to gambling activities and gamblers who have unsecured debt often take on new loans [37]. In addition, gambling-related debt tends to be more psycho-socially burdensome (e.g., strain social relationships or harm wellbeing) than non-gambling-related yet problematic debt [37, 38]. Yet, some studies have found no association between problem gambling severity and financial practices [39]. Hence, despite the growing number of studies on gambling, there is a gap in research literature that would investigate the role of consumer credit and debt problems on psychological harms caused by gambling. This type of investigation calls for multinational understanding, as the burden of gambling debt may vary from country to country.

There is significant variation between different countries in how insolvency is handled and regulated. Nordic countries, such as Finland or Sweden, take the pro-creditor stance [4, 40]. In Finland, for instance, personal bankruptcy is not possible, and debt remains enforceable for 15 years in commercial cases and 20 years in criminal cases. Debt adjustment is rare and possible only when well justified, such as in the case of illness or business bankruptcy, and in the absence of crime or suspicious economic activity [8, 41]. This means that personal misjudgments on financial decisions can lead to long-term consequences that affect all other forms of financial activities as well. In Nordic countries, debts are typically enforced by the state. A register-based study showed, to illustrate, that in Finland, one fifth of the population had debts or fines enforced during 2005–2013 [42]. In the USA, individuals have a better chance for a “fresh start” after personal bankruptcy [43]. These differences highlight the importance of better understanding consumer credit taking from cross-national perspective and considering the psychological consequences of these problems; when impacted at a young age debt problems have been associated with psychological distress, anxiety, depression, shame, and suicidal ideation [44–47]. However, US studies have also linked both student debt and credit card debt with higher sense of mastery and self-esteem among young adults. This is especially true among young people with lower or middle-class background, implying that access to even borrowed money may appear as an empowering factor or investment in the future by young adults [48].

This cross-national study sets consumer debt as a starting point for understanding potential psychological distress caused by problem gambling. In this study, psychological distress is defined as an unpleasant mental state involving symptoms of both depression and anxiety [49, 50]. The starting point is that both problem gambling and financial issues are related to psychological distress. Consumer debt provides resources for gambling which may further lead to debt problems (e.g., debt default or enforcement). We expected that consumer debt and debt problems mediate the relationship between problematic gambling and psychological distress. The role of consumer debt may, however, be context dependent. As indicated by earlier studies, mixed findings on its role on psychological distress have been reported, especially among young people in transition to adulthood [30, 44, 46, 48]. Thus, we expect that the findings are likely to vary between different contexts. Two consumer debt-orientated countries that have high gambling rates are used as examples in this study. Both the USA and Finland have easy access to consumer debt, but Finland sanctions more strictly those who are not coping well with debt. Hence, we hypothesized that consumer debt is associated with psychological distress in Finland, but not in the USA.

## Methods

### Participants

Study 1 used a demographically balanced sample of Finnish participants aged 18 to 25 years (*n* = 985, 50.76% female, mean age = 22.2, SD = 2.19) and was collected in March–April 2017. The respondents were recruited from a pool of volunteer respondents provided by Survey Sampling International. Data was set to mirror the Finnish population in terms of age, gender, and residential area structure. Comparing the sample with the population showed only minor deviations in terms of standard sociodemographic factors and, for these reasons, analytical weights were not applied. The comparison of the sample with current population estimates is shown in Appendix 1 [51].

Study 2 used a convenience sample collected from Finnish discussion forums and social networking sites in April–June 2017. Participants consisted of 18- to 29-year-olds (*n* = 205, 54.63% female, mean age = 24.36, SD = 2.89). The selected discussion forums and social networking sites were some of the most popular ones among Finnish young people and young adults. Participants were recruited by providing a short invitation and a survey link on a message board. To ensure their visibility, the invitations were activated on a regular basis during the data collection period. Males and immigrants are slightly underrepresented in this sample while people living in the Helsinki capital region are overrepresented (see Appendix 1). Analytical weights were not applied due to the convenience sampling and relatively small sample size. In addition, the main purpose of study 2 was to test whether results from study 1 replicate with a different sampling technique.

Study 3 was based on a demographically balanced sample of Americans aged 18 to 25 years (*n* = 883, 50.17% female, mean age = 21.54, SD = 2.36). This sample was collected in January 2018. Like study 1, this sample also used a pool of respondents provided by Survey Sampling International. Data was found to mirror the US population aged 18 to 25 years in terms of age, gender, and geographical area. Participants entered the study from 50 different states, with highest response rates coming from California (12.51%) and New York (7.39%), Texas (6.37%), Pennsylvania (5.35%), and Florida (5.01%). Respondents were 56.17% white alone (not Hispanic), 18.01% Hispanic, 12.91% black or African American, and 9.51% Asian. 94.66% of the respondents had a high school degree. These figures are close to the current population estimates (see Appendix 2) [52, 53]. Weights were not applied due to the close resemblance to the population estimates.

### Procedure

All three studies were part of a comparative research project on gambling among young people. The surveys were conducted with LimeSurvey software, and they were optimized for both computers and mobile devices. All surveys were run with the University server. Studies 1–3 were identical in layout and order of questions. The questionnaires for studies 1 and 2 were in Finnish, and they were translated into English for study 3 purposes and back-translated to guarantee the accuracy and matching with the original Finnish survey. The surveys were pre-tested with University students and Mechanical Turk respondents.

The study format was approved by The Academic Ethics Committee of the Tampere Region in Finland (decision 62/2016)*.* All participants agreed to voluntarily take part in the study and they were informed about the aims of the study. They had the possibility to withdraw, totally or partially, from the survey at any time during the completion process. The participants were also provided with information on how to follow the progress of the study. The data collection ensured the anonymity of the participants and the datasets were de-identified after the data collection.

The median survey response time was 920 s (15.33 min) in study 1 and 1062 s (17.70 min) in study 2. Study 3 had a median response time of 875 s (14.58 min). Additional data quality checks were run with both response time and attention check questions included in the questionnaire. In these online surveys, each question was set to mandatory so that it was not possible to proceed without answering all the questions. Hence, the surveys do not include missing data. The samples used in this article include only those respondents who responded to the measures applied in the analysis.

### Measures

Psychological distress was measured with the widely used 12-item General Health Questionnaire (GHQ-12). It evaluates the current state of psychological wellbeing with questions such as “have you recently felt constantly under strain” [49, 54, 55]. The scale has been found consistent in a number of previous studies with good to excellent internal consistency and also good construct validity over time [54, 56, 57]. The scale had good internal consistency in all three studies with Cronbach’s alphas ranging from .88 to .92 (see Table 1 for details). In population studies, Likert scoring (0-1-2-3) was applied [54, 58], and the scale ranged from 0 to 36, with higher scores indicating higher psychological distress (*M* = 3.71; SD = 3.52).

**Table 1:** Descriptive statistics. Continuous variables are presented as means (M) and standard deviations (SD). Categorical variables are presented as frequencies (*n*) and relational proportions (%)

	Study 1 (*n* = 985)				Study 2 (*n* = 205)				Study 3 (*n* = 883)			
	Finland nationwide				Finland social media				US nationwide			
Continuous variables	Range	M	SD	*α*	Range	M	SD	*α*	Range	M	SD	*α*
Psychological distress	0–36	14.43	6.35	0.88	0–36	15.62	7.14	0.92	0–36	14.53	6.99	0.88
Problem gambling (SOGS)	0–20	1.67	2.59	0.89	0–20	2.13	3.25	0.84	0–20	1.51	2.77	0.90
Problem gambling (SOGS-M)	0–10	1.52	2.10	0.82	0–10	1.84	2.37	0.86	0–10	1.25	2.04	0.83
Age	18–25	22.19	2.19	–	18–29	24.36	2.89	–	18–25	21.54	2.36	–
Categorical variables	Coding	%	*n*		Coding	%	*n*		Coding	%	*n*	
Gender	Male	49.24	485		Male	45.37	93		Male	49.83	440	
	Female	50.76	500		Female	54.63	112		Female	50.17	443	
Consumer debt	No	85.58	843		No	72.68	149		No	87.43	772	
	Yes	14.42	142		Yes	27.32	56		Yes	12.57	111	
Debt problems	No	94.72	933		No	90.24	185		No	68.97	609	
	Yes	5.28	52		Yes	9.76	20		Yes	31.03	274	

Problem gambling was measured with the South Oaks Gambling Screen (SOGS). The SOGS has been widely used in both Finland and the USA to measure problem gambling [59–62]. The SOGS reviews gambling activities from the past 12 months and scrutinizes factors indicating potential gambling problems. In population studies, internal consistence has ranged from .69 to .92 [63]. The scale had good reliability in all three studies ranging from .84 to .90. The SOGS score ranges from 0 to 20 and higher scores indicated problem gambling. The results section reports the SOGS score as categorical. DSM-V criteria and a cutoff of ≥ 8 points were used as criteria for probable disordered gambling [64]. Since borrowing money has been considered as a symptom for problem gambling, the SOGS includes the questions “Have you ever borrowed from someone and not paid them back as a result of your gambling?” and “Have you ever borrowed or acquired money to gamble or to pay gambling debts?” Due to this, separate analyses were run by creating a modified SOGS-scoring (SOGS-M) that omits the loan options. This variable ranged from 0 to 10 and had good reliability. Results based on both types of scoring are reported in the article.

Consumer debt was measured in the Finnish surveys with a question “Have you ever taken instant loans, payday loans or consumer credit?” Answer options were yes and no. The US survey asked first whether respondents had taken a loan and then specified the type of loan taken: personal loan, consumer or credit card loan, cash advance loan, and payday loans were categorized as consumer credit. Dummy variable was created (0 = no consumer debt, 1=consumer debt).

Debt problems were screened in the Finnish survey with the question “have you ever had your debt been enforced.” The Finnish word “ulosotto” refers to the enforcement process and it is explicit to the Finnish respondents. Within the Finnish system, creditors can request a court statement to start enforcement, after which the Finnish state enforcement authorities will take care of the debt. Having been to debt enforcement is an indicator of serious financial difficulties in Finland [65, 66]. Followed by the question on debt enforcement, the respondents were asked (a) whether they have taken care of their enforced debt, (b) whether they are still actively paying it back, or (c) whether they are currently considered as temporarily insolvent by the state. Those respondents who indicated having active debt problems (option b or c) were considered as having debt problems (0 = no debt problems, 1=debt problems). In the US survey, the respondents were asked “Have you ever experienced financial hardships that caused you default on payments, impacted your credit score, or got sent to collection agencies?” The response options provided were yes and no. This question was operationalized as debt problems (0 = no, 1 = yes).

### Statistical analyses

**Fig. 1:**
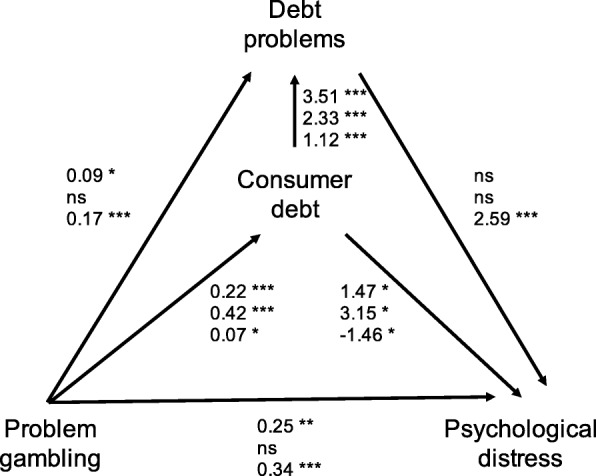
Path diagram on the role of problem gambling, consumer debt, and debt problems on psychological distress. Note: Coefficients for each path are shown: study 1 (top), study 2 (middle), and study 3 (bottom)

The statistical analyses of this article were based on descriptive statistics and generalized structural equation models (GSEM) that were fit with Stata15. The path for psychological distress was implemented by using a linear regression model with Gaussian distribution. Paths to both instant loans and debt problems are based on logistic models with Bernoulli distribution. Akaike Information Criterion (AIC) and Bayesian Information Criterion (BIC) were used to find the best model fit. GSEM coefficients are reported in the tables (Table 2 and Appendix 3) and in a path diagram (Fig. 1).

**Table 2:** Generalized structural equation models, regression coefficients and standard errors (SE), and statistical significances (*p*)

	Study 1			Study 2			Study 3		
	Finland nationwide			Finland social media			US nationwide		
	Coeff.	SE	*p*	Coeff.	SE	*p*	Coeff.	SE	*p*
Psychological distress									
Consumer debt	1.47	0.66	0.025	3.15	1.31	0.016	− 1.46	0.70	0.038
Debt problems	− 0.80	1.01	0.427	− 1.25	1.80	0.487	2.59	0.51	0.000
Problem gambling	0.25	0.08	0.002	0.11	0.18	0.526	0.34	0.08	0.000
Consumer debt									
Problem gambling	0.22	0.03	0.000	0.42	0.08	0.000	0.07	0.03	0.022
Debt problems									
Consumer debt	3.51	0.39	0.000	2.33	0.64	0.000	1.12	0.21	0.000
Problem gambling	0.09	0.04	0.032	0.09	0.06	0.134	0.17	0.03	0.000
Indirect effect (consumer debt)	0.33	0.15	0.032	1.33	0.61	0.029	− 0.10	0.07	0.123
Indirect effect (debt problems)	− 0.08	0.10	0.456	− 0.11	0.18	0.529	0.44	0.11	0.000
Model *n*	985			205			883		
AIC	7461.55			1688.92			7588.00		
BIC	7510.47			1722.15			7635.83

A mediation analysis is used in this article to provide additional details for the analyses based on GSEM. This analysis was conducted by using the *medeff* command in Stata [67]. This statistical package follows the general procedure for mediation analysis [68] but allows logistic modeling for binary mediators [67]. This study used both consumer debt and debt problems as mediators and controlled for age and gender to guarantee the robustness of the findings. The outcome variable was psychological distress, and both the SOGS and SOGS-M were used as independent variables. Mediation was run with 1000 simulations using quasi-Bayesian Monte Carlo approximation of parameter uncertainty. Total mediated effects and average causal mediation effects (ACME) are reported in the tables.

## Results

Problem gambling is a prevalent phenomenon in both Finland (studies 1 and 2) and the USA (study 3). Table 1 reports the descriptive statistics for the SOGS-scores. Prevalence of probable gambling disorder (SOGS score ≥ 8) was 3.96% in study 1, 7.80% in study 2, and 4.42% in study 3. Mean scores for psychological distress were 14.43 (study 1), 15.52 (study 2), and 14.53 (study 3). These GHQ-12 scores were correlated with the SOGS-scores in all studies, although rather weakly: study 1 *r* = .12, *p <* .001; study 2 *r* = 0.13, *p* = .059; and study 3 *r* = .22, *p <* .001.

Consumer debt taking was prevalent in all three studies: 14.42% (study 1), 27.32% (study 2), and 12.57% (study3). Debt problems were reported by 5.28% (study 1), 9.76% (study 2), and 31.03% (study 3) of the respondents. Studies 1 and 2 showed a strong association between consumer debt and debt problems. In study 1, 30.28% of respondents who had taken consumer debt had debt problems, while only 1.07% of those without consumer debt had debt problems. The respective figures were 28.57% and 2.68% for study 2 and 55.36% and 27.55% for study 3.

Comparison of means based on a two-sample *t* test showed that those with consumer debt reported higher GHQ-12 scores (i.e., higher psychological distress) than those with no consumer debt in study 1 (15.91 vs. 14.18; *p* = .003) and study 2 (17.96 vs. 14.74, *p = .*004), but not in study 3 (14.05 vs. 14.58; *p =* .46). Respectively, those with debt problems reported higher psychological distress compared to those without debt problems. However, these results were not statistically significant in study 1 (15.23 vs. 14.38 vs., *p* = 0.35) and study 2 (16.50 vs. 15.52, *p* = 0.56). In study 3, those with debt problems had higher psychological distress than those with no debt problems (16.48 vs. 13.63, *p <* .001).

The GSEM was run on a theory-based model and the estimated coefficients and associations are reported in Table 2 and Fig. 1. First of all, we found that in both countries, problem gambling is associated with consumer debt, which is further associated with debt problems. The mediating effect of consumer debt on debt problems was statistically significant in all three studies: study 1 (*p* < .001), study 2 (*p* = .002), and study 3 (*p* = .028). Problem gambling was associated with psychological distress only in studies 1 and 3. The model generally underlines the mediating function of both consumer debt and debt problems on psychological distress. In studies 1 and 2, problem gambling is associated with consumer debt, which has a positive association with both debt problems and psychological distress. Debt problems were not, however, associated with psychological distress. Consumer debt mediated the relationship between problem gambling and psychological distress in both study 1 (*p =* .049) and study 2 (*p* = .045). Study 3 shows that in the USA, it is rather the debt problems that have an association with psychological distress. The mediating effect of debt problems on psychological distress had statistical significance (*p* < .001). Interestingly, consumer debt was negatively associated with psychological distress. Running the GSEM with alternative scoring for problem gambling (SOGS-M) did not cause major deviations in the results (see Appendix 3).

Additional analyses were run to estimate the mediating effect of both consumer debt and debt problems on psychological distress. These analyses were run separately with two problem gambling variables (SOGS and SOGS-M) as independent variables (see Table 3). Gender and age were adjusted in the analysis. Consumer debt mediated strongly the relationship between problem gambling and psychological distress in study 1 (5% SOGS and 4% SOGS-M of total effect being mediated) and study 2 (49% SOGS and 31% SOGS-M of total effect being mediated), but not in study 3. Debt problems did not have a mediation effect in studies 1 and 2. Study 3 showed that in the USA, debt problems mediated the relationship between problem gambling and psychological distress with 5% (SOGS) and 9% (SOGS-M).

**Table 3:** The mediating effect of consumer debt and debt problems on psychological distress, mediated total effects, and average causal mediation effects (ACME)

	Study 1		Study 2		Study 3	
	Finland nationwide		Finland social media		US nationwide	
Mediator	SOGS	SOGS-M	SOGS	SOGS-M	SOGS	SOGS-M
Consumer debt						
Mediated total effect (%)	5.35%	4.42%	48.99%	31.19%	− 0.23%	− 0.47%
ACME	0.02	0.03	0.26	0.24	0.00	0.00
Debt problems						
Mediated total effect (%)	0.03%	0.08%	− 0.32%	− 0.30%	5.13%	9.40%
ACME	0.00	0.00	0.00	0.00	0.02	0.06

## Discussion

Three studies investigated the mediating effect of consumer debt and debt problems in Finland and the USA among emerging adults. The findings indicated, as expected, that problem gambling was directly associated with both consumer debt and debt problems. Also, studies 1 and 3 showed a direct association between problem gambling and psychological distress. Consumer debt was also linked to debt problems in all three studies. Hence, our findings suggest that problem gambling is related to consumer debt, and the consumer debt has a role in making the severe financial problems worse. Consumer debt also mediated the relationship between problem gambling and psychological distress in studies 1 and 2 in Finland. The results based on study 3 underlined the mediating role of debt problems in the USA.

The findings highlight the impact contextual differences have on debt taking and their subsequent outcomes. In Finland, debt problems are almost always linked with consumer debt. Consumer debt among young people in Finland often actualizes as payday loans (or instant loans) which tend to become very expensive, very quickly for individuals [1, 42, 69]. Furthermore, the Finnish system punishes individuals who do not take care of their debt. During the enforcement process, individuals must pay interest to the creditor. In law, this is set to be 7% higher than the benchmark interest rate. Studies show that, in Finland, the risk of being enforced increases dramatically after the age of 18, and it is quite common among 18- to 25-year-olds [8, 11]. Hence, it is perhaps no surprise that consumer debt is also a major stressor in Finland. This study reflects previous findings from other countries that underline that consumer credit especially, is associated with psychological distress [70]. A peculiarity of the Finnish system is that it is the consumer debt itself, rather than the eventual debt enforcement, that is related to psychological distress. This may be due to the fact that, after young people have lost control of their finances and ended up in debt enforcement, their financial issues are also handled by the enforcement officials.

In the USA, on the contrary, consumer debt was negatively associated with psychological distress, meaning that those who had taken consumer debt were less distressed. This finding is in line with some previous studies in the USA, showing that debt is not necessarily only a negative thing in the USA, but it may also facilitate empowerment or sense of control [43]. In the USA, consumer debt was, however, associated with higher debt problems which were, in turn, associated with psychological distress. This implies that wellbeing may be supported by borrowed money but only if the debt is in manageable degree. Overall, our findings imply that debt is a significant factor in gambling, and it is further associated with decreased wellbeing among young problem gamblers. A further challenge arises when young individuals do not have the necessary tools or resources to cope with the difficulties at hand.

Currently, young people globally face situations where different types of loans are widely available. At the same time, gambling is more popular than ever and, similarly, widely available and promoted, both locally and globally via the Internet [26]. Consumer debt and gambling are potentially a very worrying combination for emerging adults who are only learning economic independence and monetary management. Emerging adulthood is to this day characterized with impulsivity, short-sighted decision making, and instability [12]. Different loan sharks and predatory operators are also aware of this and willing to take advantage of the inexperienced young individuals [1, 69]. Especially in the pro-creditor type of systems, such as in Finland [40, 41, 43], the potential risks of careless financial management are solely the responsibility of the young people themselves.

Our results imply that it would be important to regulate both gambling and payday loans during emerging adulthood. More vigorous regulations on gambling are important due to the increased popularity of gambling among youth in many countries [22, 24, 25]. This is challenging as gamblers may use foreign gambling sites which are not following any consumer protection guidelines or age restrictions. A recent Australian study, for example, showed that one quarter of online gamblers had used offshore sites and those were also the ones with more severe gambling problems [71]. In the Finnish online gambling sites controlled by the national betting agency Veikkaus, age of the users is regulated by demanding identification with one’s personal bank account login credentials. However, this kind of regulation is missing in many foreign gambling sites, making them attractive and easy to access for underage individuals.

Regulating credit is another policy measure. Concerns have arisen that, due to cooperation between gambling and payday companies, gaming sites offer direct advertising to different lending outlets, thus increasing their visibility to gamblers. A policy recommendation suggests that these should be discouraged [72]. In addition, regulating predatory lending practices and interest rates is important. For example, the Finnish law regulates only the interest rates of loans below 2000 Euros. Due to this, many loan companies offer expensive loans above this set limit. Also, a positive credit reporting system would provide more information about credit history and debt management and might hence function as an effective preventive measure, especially if creditors are supervised and expected to avoid lending for those with existing financial difficulties [42]. Currently, the Finnish system does not give this information to the creditors, although similar systems have been employed in many other countries, including the USA and several EU countries.

This study was limited by its cross-sectional design and self-reported data which do not enable us to determine the impact of consumer debt and debt problems on the long run. Study 2 was collected via social networking sites and the sample was relatively small when compared to the nationwide samples in studies 1 and 3. The sample in study 2 was also a convenience sample collected from social networking sites and it might have attracted more respondents generally interested in gambling. The study is also limited by the use of non-standardized measurement of consumer debt and debt problems. Due to the legal and societal differences, the used measures were slightly different in the USA (study 3) and Finland (studies 1 and 2). Despite these limitations, all three studies had major strengths, including widely tested measures for problem gambling and psychological distress. We were also able to demonstrate the key findings by using two different statistical solutions. This allows for more robust interpretation of our key findings.

## Conclusion

The results suggest that problem gambling-related psychological distress is partly explained by consequent consumer debt or debt problems. The role of consumer debt should be also considered in the context of gambling pathology. Although loss of money and property is commonly accompanied by problem gambling, the current theories have not considered far enough how addictions and consumerism go hand in hand in Western debt societies. For gamblers, consumer credit and payday loans provide resources that enable them to keep up with the gambling habit, even in situations where significant amounts of money have been lost due to the behavior. This may eventually lead to debt problems and psychological distress. The results imply that limiting consumer debt by law and regulation would be important as practical means and they could cushion the potentially far-reaching financial and psychological costs of youth problem gambling.

## Appendix 1

**Table 4:** Finnish samples (study 1 and study 2) vs. population

	Study 1 (Fin)	Study 2 (Fin)	Population (Fin)
	%	%	%
Male	49.24	42.19	51.33
Residential area			
Helsinki area	26.50	32.00	27.94
Other towns or cities	62.28	58.40	61.15
Countryside	11.21	9.60	10.90
Upper secondary degree	87.22	80.21	76.16
Foreign born	4.37	3.12	7.43

Note: Population figures are based on Statistics Finland information [51]. Information on education and land of birth are for 20–24-year-olds. The rest of the figures are for 18–25-year-olds

## Appendix 2

**Table 5:** US sample (study 3) vs. population

	Study 3 (USA)	Population (USA)
	%	%
Male	49.83	51.31
US state		
California	12.51	12.14
New York	7.39	6.09
Texas	6.37	8.69
Pennsylvania	5.35	3.93
Florida	5.01	6.44
High school degree	94.66	92.97
Foreign born	5.66	9.76
White	56.17	54.18
Hispanic	18.01	21.76
African American	12.91	14.58

Note: Population figures are based on US Census Bureau Statistics for the year 2016 [52, 53]. All the figures are for 18–24-year-olds

## Appendix 3

**Table 6:** Alternative generalized structural equation models using SOGS-M for problem gambling, regression coefficients and standard errors (SE), and statistical significances (*p*)

	Study 1			Study 2			Study 3		
	Finland nationwide			Finland social media			US nationwide		
	Coeff.	SE	*p*	Coeff.	SE	*p*	Coeff.	SE	*p*
Psychological distress									
Instant loans	1.33	0.65	0.042	2.80	1.32	0.033	− 1.46	0.70	0.037
Debt problems	− 0.81	1.01	0.421	− 1.29	1.78	0.471	2.55	0.52	0.000
Problem gambling (SOGS-M)	0.39	0.10	0.000	0.29	0.24	0.228	0.46	0.11	0.000
Consumer debt									
Problem gambling (SOGS-M)	0.29	0.04	0.000	0.49	0.08	0.000	0.10	0.04	0.016
Debt problems									
Instant loans	3.52	0.39	0.000	2.42	0.64	0.000	1.12	0.21	0.000
Problem gambling (SOGS-M)	0.12	0.06	0.054	0.09	0.09	0.299	0.24	0.04	0.000
Indirect effect (consumer debt)	0.38	0.20	0.049	1.38	0.69	0.045	− 0.15	0.10	0.115
Indirect effect (debt problems)	− 0.10	0.13	0.457	− 0.12	0.20	0.554	0.61	0.15	0.000
Model *n*	985			205			883		
AIC	7452.96			1691.49			7582.09		
BIC	7501.89			1724.72			7629.92		

## Abbreviations

AIC: Akaike Information Criterion
BIC: Bayesian Information Criterion
GSEM: Generalized structural equation models
M: Mean
SE: Standard error
SOGS: South Oaks Gambling Screen, refers to the score provided by the screener
SOGS-M: Modified score based on shortened SOGS
